# Limited contribution of natural killer cells to *SOD1*^*G93A*^ ALS pathogenesis: evidence from comprehensive immunophenotyping and genetic deletion of *Ncr1* and *Properdin*

**DOI:** 10.1016/j.bbih.2026.101401

**Published:** 2026-11-05

**Authors:** Roxane Crabé, Alexandre Brodovitch, Philippe Gosset, Charlotte Gavard, Véronique Brugioti, Désiré Challuau, Antoine Picot, Gabriel Espinosa-Carrasco, Laurent Soustelle, Kevin Mouzat, Javier Hernandez, José Boucraut, Thierry Vincent, Cédric Raoul

**Affiliations:** a INM, Univ Montpellier, INSERM, Montpellier, France; b Reference Centre for ALS and Neuromuscular diseases, La Timone University Hospital, Aix-Marseille University, Marseille, France; c Biogenopole Laboratory, AP-HM, La Timone University Hospital, Marseille, France; d IRMB, Univ Montpellier, Montpellier, France; e Laboratory of Biochemistry and Molecular Biology, CHU Nimes, Univ Montpellier, Nîmes, France; f Aix Marseille Univ, INS, INSERM, UMR 1106, Marseille, France; g Laboratory of Immunology, Univ Montpellier, CHU Montpellier, Montpellier, France; h ALS Reference Centre, Univ Montpellier, CHU Montpellier, Montpellier, France

**Keywords:** Amyotrophic lateral sclerosis, Neuroimmunity, Natural Killer cells, Lymphocytes, Natural cytotoxicity triggering receptor 1, Properdin, Motoneurons

## Abstract

Immune dysregulation contributes to amyotrophic lateral sclerosis (ALS) pathogenesis. Natural killer (NK) lymphocytes have been proposed to contribute to ALS, but their exact role remains debated. Here we combined comprehensive immunophenotyping strategy with two complementary loss-of-function approaches to trace NK cells and assess their Ncr1-associated cytotoxic function in *SOD1*^*G93A*^ mice. We found no significant difference in the proportion, maturation, and activation status of circulating NK cells between control and *SOD1*^*G93A*^ mice at onset and symptomatic stage, and no significant NK cell recruitment to the central nervous system at any disease stage. Notably, resident microglia in the *SOD1*^*G93A*^ spinal cord acquire NK1.1 expression at the symptomatic stage. NK cells isolated from *SOD1*^*G93A*^ mice did not affect motoneuron survival *in vitro*. We further show that genetic deletion of *Ncr1*, which abolishes one of the major activating pathways of NK-cell cytotoxicity, did not modify disease onset, motor function or survival of *SOD1*^*G93A*^ mice. Additionally, we show that Properdin, a positive regulator of the alternative complement pathway and a reported ligand of NCR1, decorates misfolded SOD1 inclusions and degenerating astrocytes, consistent with a role as a phagocytic signal for CD68^+^ microglia. However, genetic deletion of *Properdin* in *SOD1*^*G93A*^ did not influence neuroinflammation or motoneuron survival. Together, the unchanged frequency, maturation or activation status of NK cells, combined with the lack of effect of *Ncr1* genetic ablation argue against a major NK contribution to motoneuron degeneration in the *SOD1*^*G93A*^ mouse model, while uncovering an unexpected role for Properdin in opsonizing misfolded SOD1 aggregates.

## Highlights

• NK cell frequency, maturation and activation are unchanged in the blood of SOD1^G93A^ mice.
• NK cells are not significantly recruited to the CNS of SOD1^G93A^ mice.
• NK cells from ALS mice do not affect motoneuron survival *in vitro*.
• Ncr1 deletion does not alter disease onset, motor decline or survival in ALS mice.
• Properdin opsonizes misfolded SOD1 inclusions and degenerating astrocytes in SOD1^G93A^ mice.

## 1 Introduction

Amyotrophic lateral sclerosis (ALS) is a fatal, progressively paralyzing neurodegenerative disorder affecting motoneurons in the motor cortex, brainstem and spinal cord, with a typical survival of 3 years post-diagnosis (Masrori and Van Damme, 2020). ALS affects both upper and lower motoneurons residing in the motor cortex, brainstem and spinal cord. The incidence of ALS ranges from 2 to 3 per 100 000 person-years, with an age of onset between 50 and 70 years (Longinetti and Fang, 2019). While most ALS cases are sporadic, approximately 10% of patients have a familial history of the disease (Mathis et al., 2019). Mutations in Cu-Zn superoxide dismutase (SOD1) were the first identified genetic causes and are among the most frequently found in familial ALS. Transgenic mice overexpressing SOD1 mutations exhibit key aspects of ALS pathology and clinical course and remain an essential model for comprehending the underlying pathogenic mechanisms. Beyond motoneuron degeneration, ALS is typified by an overt glial response, which gain detrimental functions as disease progresses (Haukedal and Freude, 2019; Yamanaka and Komine, 2018). An immune response has also recently been recognized as an important pathological contributor to disease progression (Bowerman et al., 2012; Chiot et al., 2019). Regulatory T cells (Treg) which are pivotal in suppressing immune responses, increase at the early stage of the disease, then rapidly decrease at the progression phase, as their suppressive capacity becomes reduced (Beers et al., 2017). The adoptive transfer of Treg can prolong survival in ALS mice (Beers et al., 2011). We and others have shown that CD8^+^ T lymphocytes infiltrate the central nervous system (CNS) in both ALS patients and mice (Chiu et al., 2008; Coque et al., 2019; Engelhardt et al., 1993). CD8^+^ T cells expressing SOD1 mutant can selectively kill motoneurons that are presumably electrically silent. The ablation of CD8^+^ T cells resulted in the reduction of motoneuron loss in ALS mice (Coque et al., 2019).

The contribution of Natural Killer (NK) lymphocytes to ALS pathogenesis remains ambiguous. Unlike CD8^+^ T cells, NK cells exert cytotoxic activity independent of antigen presentation by major histocompatibility complex (MHC). Their activity depends on a balance of inhibitory signals provided by any cell expressing self-MHC class I (MHC-I) and stimulatory signals when transformed cells lose expression of MHC-I or express other stimulatory molecules (Vivier et al., 2011). The number of NK cells, based on CD3, CD16 and CD56 expression, has been reported to increase in the peripheral blood of patients with ALS, compared to healthy individuals (Gustafson et al., 2017; Jin et al., 2020; Murdock et al., 2017; Rentzos et al., 2012). However, a reduction of CD3^−^CD56^+^ cells has been reported in the peripheral blood of sporadic ALS patients compared to control (Garofalo et al., 2020). Although NK cell number has been reported to be inversely correlated with ALS functional rating scale (ALSFRS-R)(Gustafson et al., 2017). NK cell changes were not directly correlated with changes in ALSFRS-R scores (Murdock et al., 2017). Another study documented a significantly higher amount of CD56^bright^CD16^dim^ NK cells in slowly progressing ALS patients compared to fast ALS (Jin et al., 2020).

A significant increase of NK1.1^+^ cells has been described in the spinal cord of *SOD1*^*G93A*^ mice (Chiu et al., 2008; Garofalo et al., 2020; Komine et al., 2018). With regard to the function of NK cells in ALS pathogenesis, injection of depleting anti-NK1.1 antibody has no effect on survival. Conversely, increasing the number of infiltrating NKT and NK cells in the spinal cord of *SOD1*^*G93A*^ mice by injection of interleukin (IL)-2 complex has no effect on the lifespan of ALS mice (Komine et al., 2018). However, another study showed that injection of depleting anti-NK1.1 in *SOD1*^*G93A*^ and *TDP-43*^*A315T*^ mice delayed disease onset and extended lifespan of mice (Garofalo et al., 2020). The latter study proposed that NK cells exert a cytotoxic activity against motoneurons in the neurodegenerative context.

Here, we aimed to trace NK cells and investigate their cytotoxic role in ALS pathogenesis combining a genetic tool targeting the natural cytotoxicity triggering receptor (Ncr1) and different markers of NK cells (Gazit et al., 2006). Ncr1 (NKp46), which belongs to the same family as Ncr2 (NKp44) and Ncr3 (NKp30), is expressed on NK cells and acts as an activating receptor for non-MHC ligands expressed by target cells. Ncr1 activation triggers NK cell degranulation and cytotoxicity, as well as the production of proinflammatory cytokines, notably interferon gamma (IFNγ) (Pazina et al., 2017). The number, maturation, and activation status of circulating NK cells did not differ between *SOD1*^*G93A*^ and wildtype mice, and no significant NK cell infiltration was observed in the CNS, in contrast to previous reports (Chiu et al., 2008; Garofalo et al., 2020; Komine et al., 2018). SOD1^G93A^-expressing NK cells did not affect motoneuron survival in co-culture, and genetic ablation of *Ncr1* had no effect on disease onset, motor decline, motoneuron survival, or lifespan in *SOD1*^*G93A*^ mice.

To further validate our findings, we next investigated Properdin. Properdin (complement factor P) is a soluble plasma protein described as a positive regulator of complement activation, which has been proposed to act as a ligand for NKp46 (Narni-Mancinelli et al., 2017). We show that Properdin decorate misfolded SOD1 aggregates and degenerating astrocytes and may act as a phagocytic signal for microglial cells. The targeted ablation of *Properdin* gene in *SOD1*^*G93A*^ mice, however, does not influence neuroinflammation nor motoneuron survival. Our work does not support a substantial contribution of NK cells during disease progression in ALS mice.

## 2 Materials and methods

### 2.1 Animals

All animal experiments were approved by the French ministry of higher education, research and innovation (authorization 6224-2016072711103977v3), and were conducted in compliance with the European Community (directive 2010/63/EU) and national directives for the care and use of laboratory animals. B6.Cg-Tg(SOD1*G93A)1Gur/J (*SOD1*^*G93A*^) and B6; 129-*Ncr1*^*tm1Oman*^/J (*Ncr1*^*gfp/gfp*^) were purchased from the Jackson Laboratory. *Properdin* deficient mice were generated as previously described (Stover et al., 2008). All mouse strains were maintained on a C57BL/6J background under specific-pathogen-free conditions, housed in cages (8-10 animals per cage) with a 12h light/12 h dark cycle, with food and water supplied *ad libitum*. Housing conditions and experimental procedures were designed in accordance with the 3Rs principles. Mixed-sex cohorts of mice were used in all experimental groups, with littermate controls used throughout.

### 2.2 Fluorescence-activated cell sorting analysis

Mice at the symptomatic stage were euthanized and transcardially perfused with phosphate-buffered saline (PBS). Isolated spinal cords were mechanically and enzymatically dissociated and the cell suspension passed through a cell strainer. Myelin and cellular debris were removed by Percoll density gradient centrifugation. Spinal cord cell suspension and peripheral blood sample (following red blood cell lysis) were stained with the following antibody panel: anti-CD3-V500; anti-CD45-APC-Cy7; anti-CD8-V450; anti-CD49b-PerCP-Cy5.5; anti-KLRG1-PE; anti-CD11b-PE-Cy7; anti-NKp46-APC (BD Biosciences) and anti-CD69-V450; anti-NK1.1-PC7; anti-CD27-PE; anti-CD19-V500; anti-CD3-PerCP-Cy5.5; anti-CD45-APC-Cy7; anti-NKp46-APC (BD Biosciences). Data were acquired on a BD Fortessa LSRII cytometer using FACS Diva (BD Biosciences), and analyzed using FlowJo Software (BD Biosciences).

For the antibody panel used in Fig. 3F–I and Supp Fig. 4, cells were washed and stained with Zombie NIR™ Fixable Viability Kit, blocked with TruStain FcX™ PLUS antibody and then stained with the following antibodies: anti-CD45-APC; anti-CX3CR1-PE-Fire; anti-CD11b-Alexa Fluro 488; anti-CD3-PE-Cy5; anti-F4/80-BV650 and anti-NK1.1-PE antibodies from BioLegend. Data acquisition was performed on a Cytek Aurora spectral flow cytometer (Cytek) and analyzed using FlowJo Software.

For the meta-analysis of CD49b^+^ cells among CD45^+^CD90.2^−^CD3^−^ cells, the single cell suspension from the blood, spinal cord and brain of wildtype and *SOD1*^*G93A*^ mice was stained with anti-CD3-BV785 (BioLegend); anti-CD4, anti-CD45-BV650, anti-CD90.2 (Thy1.2)-V500, anti-CD8a-APC, anti-CD49b-PE-CF594, anti-CD62L-PerCP-Cy5.5, anti-CD44-FITC, anti-CD25-BV421 (BD Biosciences); and anti-KLRG1-PE-Cy7 (eBioscience) antibodies (Coque et al., 2019). The flow cytometry data were obtained using a FACSCanto II or an LSR Fortessa apparatus (BD Biosciences). The meta-analysis of the cytometry datasets was then conducted using the FACSDiva software (BD Biosciences). A summary of the antibody panels used for flow cytometry analysis, including the figures, tissue, age, markers, and cell population(s) they were designed to identify, is provided in Suppl. Table 1.

### 2.3 Functional assays

For muscle atrophy-induced weight loss, mice were weighed once a week starting at 28 days of age and three times a week from 56 days until end stage. To assess neuromuscular strength, mice were tested once a week using the grid test, starting at 56 days of age. Mice were held by the tail while gripping the grid with both their fore- and hindpaws. Starting with a 40 g metal grid, followed by 30, 20 and 10 g grids, the time the mouse held on the grid before dropping was measured, with a plateau of 30 s. The experiment was repeated three times with each grid. Muscle strength (arbitrary units) was quantified using the following formula: (40 x best time) + (30 x best time) + (20 x best time) + (10 x best time)(Saxena et al., 2009; Soulard et al., 2020). Mortality was defined as the point in time when the mice were unable to right themselves within 15 s after being placed upon their back. All these experiments were done in a blinded manner.

### 2.4 Immunohistochemistry

Mice were anesthetized and transcardially perfused with PBS and then with 4% paraformaldehyde (PFA). The spinal cord and spleen were harvested and post-fixed in 4% PFA by immersion at +4°C for 45 min. Tissues were incubated overnight in 30% sucrose solution and embedded in O.C.T. compound (Sakura Finetek). Cryosections of the spinal cord or spleen (16 μm) were collected onto Superfrost Plus slides and rinsed with PBS before being incubated for 1 h at room temperature (RT) in blocking solution (3% BSA, 3% donkey serum, 0.1% Triton X-100). Sections were incubated with goat anti-ChAT (AB144P, Merck), rabbit anti-GFP (TP-401, Torrey Pines Biolabs), rabbit anti-Properdin (ab186834, Abcam and LS-B13878, LS Biosciences), mouse anti-misfolded SOD1 (B8H10, Medimabs), rat anti-CD45 (IBL-5/25, Merck), mouse anti-CD68 (FA-11, Bio-Rad), rabbit anti-Iba1 (019-19741, Wako), mouse anti-glial fibrillary acidic protein (GFAP)(GA5, Merck), rabbit anti-cleaved caspase-3 (9661, Cell Signaling Technology), mouse anti-ubiquitin (FK2, MBL Life Science) diluted in blocking solution overnight at +4°C. After washing in PBS, sections were incubated with AlexaFluor 488, 594 or 647-conjugated donkey secondary antibodies (ThermoFisher Scientific) diluted in blocking solution for 1 h at RT. Slides were mounted in NeoBio Mount fluo solution (NeoBiotech). Images were acquired using a Zeiss LSM880 confocal microscope.

### 2.5 Motoneuron-NK cell coculture

Cervical, axillary, brachial, inguinal and mesenteric lymph nodes from wildtype or *SOD1*^*G93A*^ mice were dissected and crushed onto a nylon membrane. Total cells were then resuspended in cold PBS. NK cells were isolated by negative selection using magnetic cell isolation. The NK cell isolation kit II (Miltenyi Biotec) was used according to the manufacturer's instructions. Motoneurons from E12.5 spinal cord of wildtype or *SOD1*^*G93A*^ embryos were purified as we previously described (Soulard et al., 2020), combining iodixanol density gradient centrifugation and magnetic cell isolation (anti-CD271, clone REA648, Miltenyi). Motoneurons were plated on poly-ornithine/laminin-treated wells in the presence of neurotrophic factors including glial-derived neurotrophic factor (0.1 ng/ml), brain-derived neurotrophic factor (1 ng/ml) and ciliary neurotrophic factor (10 ng/ml) in supplemented Neurobasal medium (2% (vol/vol) horse serum, 2% (vol/vol) B-27 supplement (ThermoFisher Scientific), 50 μM L-glutamine, 25 μM L-glutamate, 25 μM 2β-mercaptoethanol and 0.5% penicillin/streptomycin). Motoneurons were seeded at the density of 1500 cells per cm^2^ and NK cells were added at the same time at the density of 15000 cells per cm^2^ (ratio 1:10). Surviving motoneurons were directly counted under phase contrast microscope 48 h later.

### 2.6 Statistical analysis

Statistical significance was determined when appropriate by unpaired two-tailed *t*-test, Kruskal-Wallis test followed by Dunn's multiple comparisons test, one-way analysis of variance (ANOVA) followed by a Tukey-Kramer *post hoc* tests or two-way repeated-measure ANOVA followed by Newman-Keuls *post hoc* test. For the Kaplan-Meier survival analysis, the Log-rank Mantel-Cox test was used. Statistical analyses were done with Prism software (GraphPad Software). Significance was accepted at the level of *P* < 0.05. The specific statistical test applied to each dataset is indicated in the corresponding figure legend.

## 3 Results

### 3.1 Circulating NK cells does not show signs of abnormal frequency, maturation or activation in ALS mice

An internal ribosome entry site followed by the green fluorescent protein (GFP) coding sequence was incorporated in the *natural cytotoxicity triggering receptor 1* (*Ncr1*) gene deleting exons 5 to 7 (Gazit et al., 2006). *Ncr1*^*gfp/gfp*^ mice, which faithfully trace NK cells, exhibit an abolished killing activity of NK cells towards target cells missing MHC-I, without affecting the development of the NK population or their migration properties (Gazit et al., 2006). We used this genetic tool to investigate the representation, maturation and activation status of NK cells in the circulating blood of ALS mice. *Ncr1*^*gfp/gfp*^ mice are viable and fertile (Gazit et al., 2006), and were bred with *SOD1*^*G93A*^ mice. To facilitate the interpretation of the experimental design, the sequence and timing of the different procedures, together with the corresponding cohorts, are summarized in a schematic timeline (Suppl. Fig. 1). To first quantify the frequency of circulating lymphocytes in wildtype, *SOD1*^*G93A*^, *Ncr1*^*gfp/gfp*^ and *SOD1*^*G93A*^; *Ncr1*^*gfp/gfp*^ mice, flow cytometry analysis with CD45, CD3, CD8 and CD19 was performed at 140 days of age (symptomatic stage). As we had previously described, the percentage of CD8^+^ T cells in the peripheral blood did not differ between wildtype and *SOD1*^*G93A*^ mice (Coque et al., 2019), nor did the percentage of CD3^+^CD8^−^ T cells and CD19^+^ B lymphocytes (Suppl. Fig. 2A–E). The frequency of these lymphocyte populations was not influenced by the loss of Ncr1 function.

To determine the percentage of circulating NK cells in ALS and littermate control mice, we combined immunolabeling of Ncr1, NK1.1 and CD49b with native GFP. Single event cells were first defined with forward scatter and side scatter parameters and the percentage of Ncr1^+^NK1.1^+^, as well as GFP^+^NK1.1^+^, determined among the CD45^+^CD3^−^ cells. As expected, we observed a complementary expression pattern between Ncr1 and GFP when mice harbor or not the *Ncr1*^*gfp/gfp*^ locus (Fig. 1A). The percentage of Ncr1^+^NK1.1^+^ cells did not vary between wildtype and *SOD1*^*G93A*^ mice, nor did the percentage of GFP^+^NK1.1^+^ NK cells between *Ncr1*^*gfp/gfp*^ and *SOD1*^*G93A*^; *Ncr1*^*gfp/gfp*^ mice (Fig. 1B and C). To confirm this observation, we use the pan-NK marker CD49b combined with Ncr1 and GFP (Fig. 1D). We did not observe however any difference in the percentage of Ncr1^+^CD49b^+^ cells between wildtype and *SOD1*^*G93A*^, or in the percentage of GFP^+^CD49b^+^ cells between *Ncr1*^*gfp/gfp*^ and *SOD1*^*G93A*^; *Ncr1*^*gfp/gfp*^ mice (Fig. 1E and F).

**Fig. 1:**
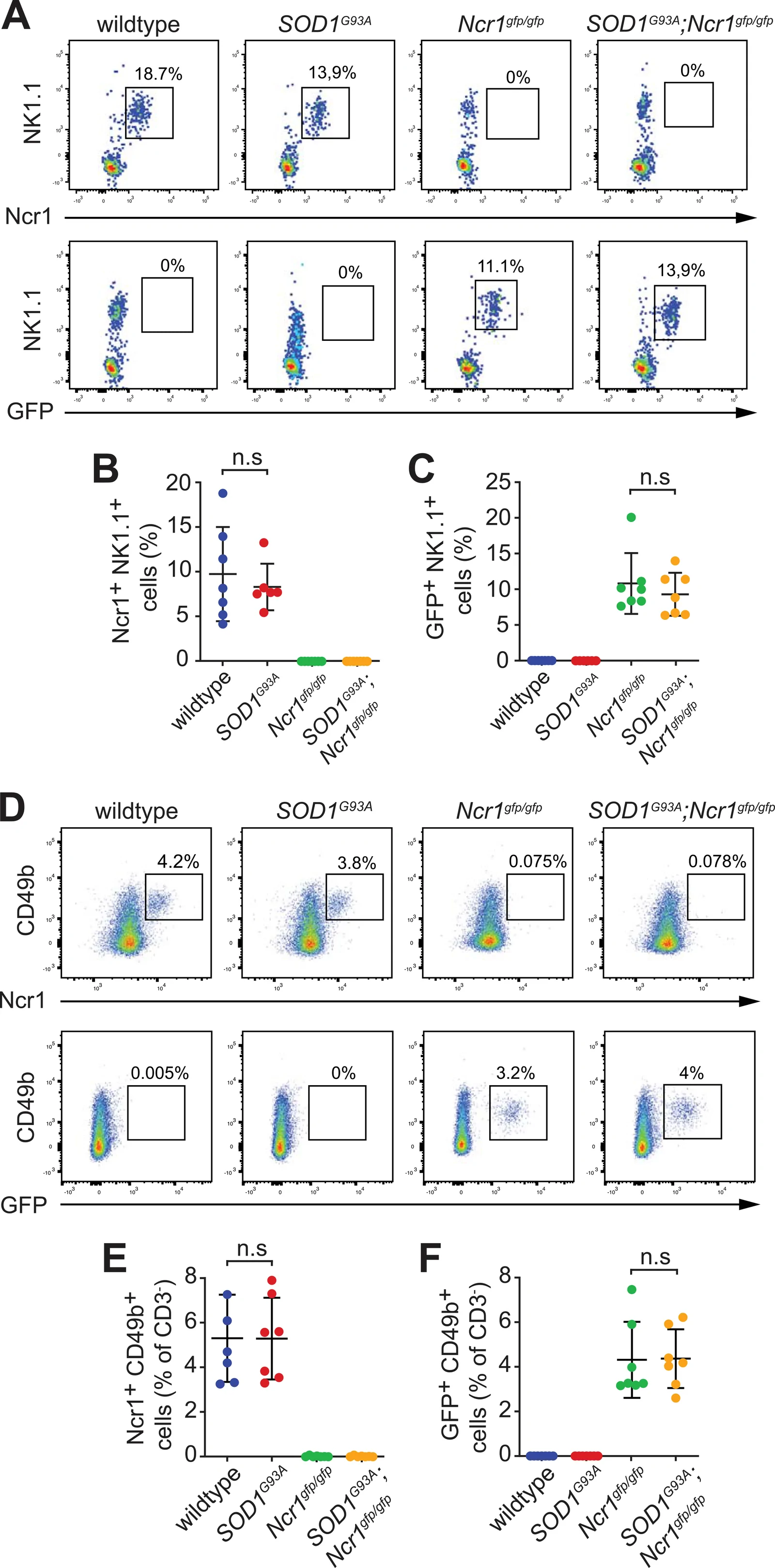
**Circulating NK cell frequency is unchanged in *SOD1*^*G93A*^ mice.** (**A**) Representative fow cytometry dot plots showing Ncr1^+^NK1.1^+^ and GFP^+^NK1.1^+^ cell populations in the peripheral blood of 140-day-old wildtype, *SOD1*^*G93A*^*, Ncr1*^*gfp/gfp*^ and *SOD1*^*G93A*^*;Ncr1*^*gfp/gfp*^ mice. (**B**,**C**) Mean frequency of circulating Ncr1^+^NK1.1^+^ (B) and GFP^+^NK1.1^+^ (C) NK cells in mice with the indicated genotype. (**D**) Ncr1^+^CD49b^+^ and GFP^+^CD49b^+^ cells in the blood of wildtype, *SOD1*^*G93A*^*, Ncr1*^*gfp/gfp*^ and *SOD1*^*G93A*^*;Ncr1*^*gfp/gfp*^ mice were identified by flow cytometry. (**E**) Percentage of circulating Ncr1^+^CD49b^+^ NK cells and (**F**) GFP^+^CD49b^+^ (F) NK cells in mice with the indicated genotype. Numbers in the quadrants indicate the percentage of gated cells, horizontal bars show mean values ± standard deviation (SD); *n* = 7 per group; n.s, non-significant; Kruskal-Wallis test with Dunn's *post hoc* test.

We next performed a meta-analysis of cytometry data obtained during our previous immunophenotyping of circulating lymphocytes in symptomatic 150-day-old *SOD1*^*G93A*^ mice (Coque et al., 2019). At this stage, CD8^+^ T lymphocytes significantly increase in the CNS, but not in the blood, of ALS mice (Coque et al., 2019). We then determined the frequency of CD49b^+^ within CD45^+^CD90.2^−^CD3^−^ cells (our previous study only included CD49b as a marker of NK cells) in blood. Coherently, we did not find any significant difference in this combination of markers between wildtype and *SOD1*^*G93A*^ mice (Suppl. Fig. 3A and B).

Nevertheless, we explored whether more discrete yet functional changes in NK subsets could exist between *SOD1*^*G93A*^ and control mice. NK precursor cells undergo a series of maturation stages that can be identified by a differential expression of surface markers (Abel et al., 2018). CD49b is expressed by mature stage D to F NK cells, CD11b by mature stage E and F NK cells, and KLRG1 by fully mature stage F NK cells. By combining CD49b, CD11b and KLRG1, we can therefore trace stage D (CD49b^+^CD11b^−^KLRG1^-^), stage E (CD49b^+^CD11b^+^KLRG1^-^) and terminal stage F (CD49b^+^CD11b^+^KLRG1^+^)(Fig. 2A). However, we did not observe any significant difference in the maturation stages of NK cells between wildtype and *SOD1*^*G93A*^ mice using the Ncr1 marker, or *Ncr1*^*gfp/gfp*^ and *SOD1*^*G93A*^; *Ncr1*^*gfp/gfp*^ mice using GFP reporter (Fig. 2B–D). This analysis was further completed by combining Ncr1, expressed from immature stage C NK cells and all mature stages; NK1.1 expressed from immature stage B NK cells and all mature stages, with CD27, which is expressed by immature and mature NK cells but whose expression is lost in mature stage F NK cells. The expression of SOD1^G93A^ in mice with or without targeted deletion of *Ncr1* did not significantly alter the maturation of NK cells (Fig. 2E and F). We then investigated the activation status of circulating NK cells in the different genotypes of mice. The C-type lectin receptor CD69 is a suitable marker to identify NK cells with cytotoxic properties (Clausen et al., 2003). We therefore compared the percentage of Ncr1^+^NK1.1^+^CD69^+^ and GFP^+^NK1.1^+^CD69^+^ NK cells between wildtype, *SOD1*^*G93A*^, *Ncr1*^*gfp/gfp*^ and *SOD1*^*G93A*^; *Ncr1*^*gfp/gfp*^, and we did not find any significant difference between different groups (Fig. 2G and H).

**Fig. 2:**
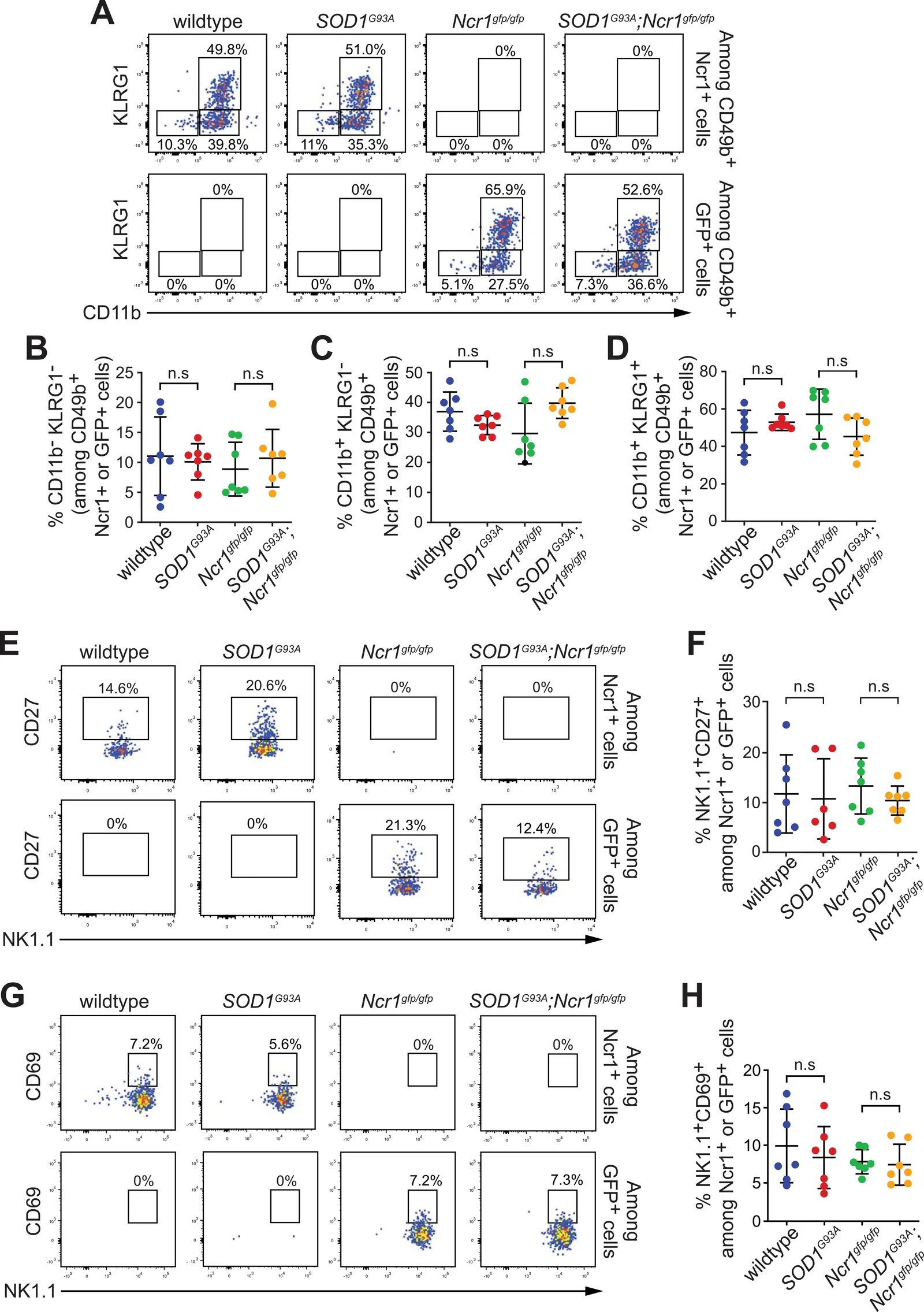
**NK cell maturation and activation status are unchanged in *SOD1*^*G93A*^ mice**. (**A**) Cells were isolated from the peripheral blood of 140-day-old wildtype, *SOD1*^*G93A*^, *Ncr1*^*gfp/gfp*^ and *SOD1*^*G93A*^; *Ncr1*^*gfp/gfp*^ mice and labeled with CD45, CD3, CD49b, Ncr1, CD11b and KLRG1. CD11b^+^ and KLRG1^+^ cells were assessed within the CD45^+^CD3^−^CD49b^+^Ncr1^+^ or CD45^+^CD3^−^CD49b^+^GFP^+^ NK cells. (**B**-**D**) Frequencies of CD11b^−^KLRG1^-^ (**B**), CD11b^+^KLRG1^-^ (**C**) and CD11b^+^KLRG1^+^ (**D**) NK cell subsets (among CD49b^+^Ncr1^+^ or CD49b^+^GFP^+^) frequencies in the peripheral blood of wildtype, *SOD1*^*G93A*^, *Ncr1*^*gfp/gfp*^ and *SOD1*^*G93A*^; *Ncr1*^*gfp/gfp*^. Data from Ncr1^+^ gates in Ncr1-deficient mice (*Ncr1*^*gfp/gfp*^ and *SOD1*^*G93A*^; *Ncr1*^*gfp/gfp*^) and GFP gates in GFP-negative mice (wildtype, *SOD1*^*G93A*^) are not shown. (**E**) Cytometry plots of CD27^+^NK1.1^+^ cells within the Ncr1^+^ or GFP^+^ NK cells of mice with indicated genotypes. (**F**) Percentages of CD27^+^NK1.1^+^ cells among circulating Ncr1^+^ or GFP^+^ NK cells from mice of the indicated genotypes. (**G**) Cytometry plots of CD69^+^NK1.1^+^ cells within Ncr1^+^ or GFP^+^ NK cells of wildtype, *SOD1*^*G93A*^, *Ncr1*^*gfp/gfp*^ and *SOD1*^*G93A*^; *Ncr1*^*gfp/gfp*^ mice. (**H**) Scatter plots showing the percentage of CD69^+^NK1.1^+^ cells among the Ncr1^+^ or GFP^+^ NK cells in the peripheral blood of mice with indicated genotypes. Representative flow cytometry plots are shown, *n* = 7. GFP expression in wildtype and *SOD1*^*G93A*^ as well as Ncr1 expression in *Ncr1*^*gfp/gfp*^ and *SOD1*^*G93A*^; *Ncr1*^*gfp/gfp*^ is not shown. Numbers in the quadrants indicate the percentage of gated cells. Bars superimposed on the scatter plots show the means ± SD; n.s, non-significant; Kruskal-Wallis test with Dunn *post hoc* test.

**Fig. 3:**
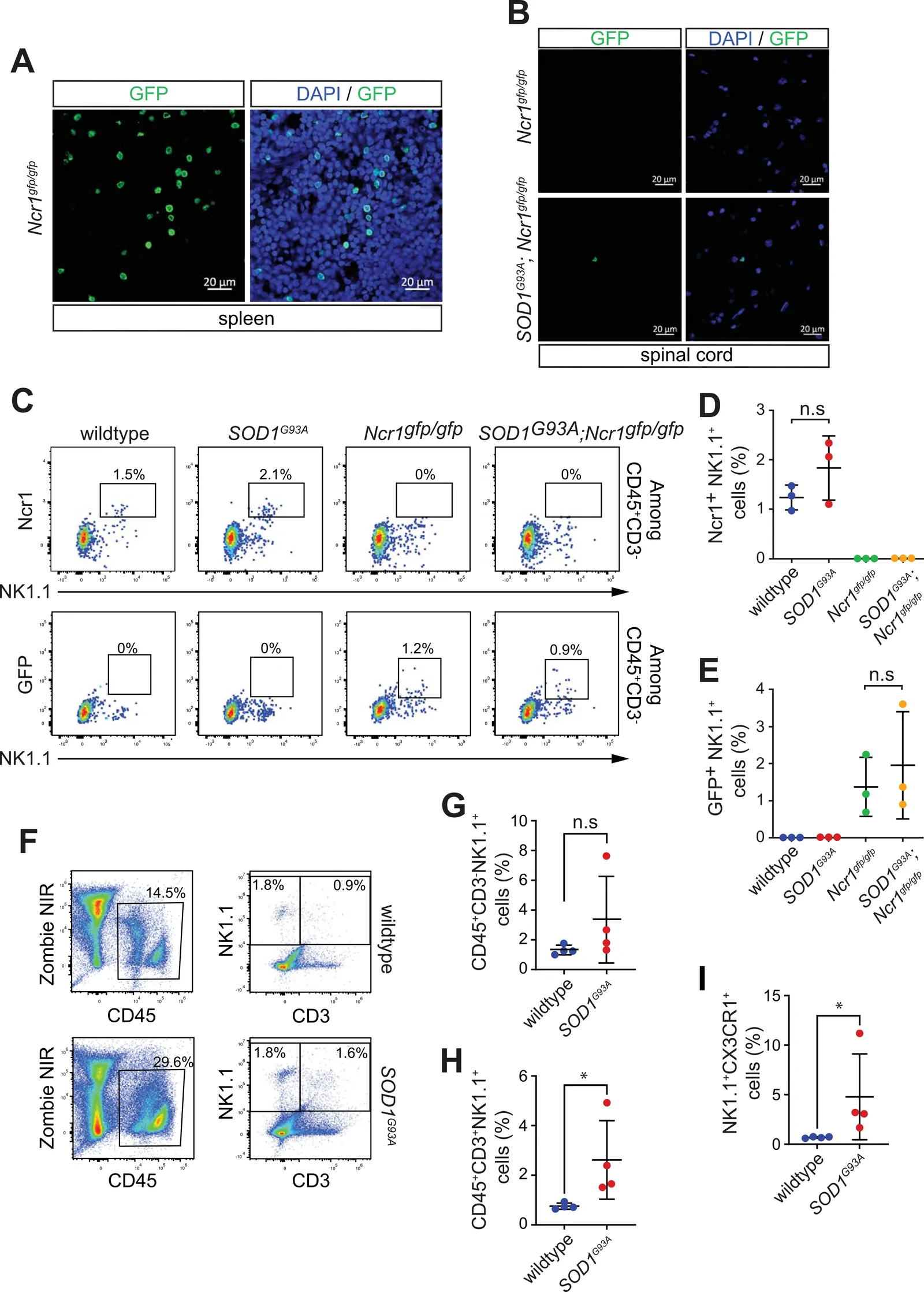
**NK cells do not significantly infiltrate the CNS of *SOD1*^*G93A*^ mice.** (**A**) GFP^+^ cells can be visualized in sections of the spleen of *Ncr1*^*gfp/gfp*^ mice. (**B**) Spinal cord sections of 140-day-old *Ncr1*^*gfp/gfp*^ and *SOD1*^*G93A*^*; Ncr1*^*gfp/gfp*^ mice were immunostained with an anti-GFP polyclonal antibody. GFP^+^ cells were observed only sporadically in the spinal cord. Scale bar, 20 μm. **(C)** Representative flow cytometry plots showing NK1.1^+^, Ncr1^+^ and GFP^+^ NK cell populations in the spinal cord of 110-day-old wildtype, *SOD1*^*G93A*^*, Ncr1*^*gfp/gfp*^ and *SOD1*^*G93A*^*;Ncr1*^*gfp/gfp*^ mice. (**D**,**E**) Percentage of NK1.1^+^Ncr1^+^ (D) and NK1.1^+^GFP^+^ (E) NK cells from wildtype, *SOD1*^*G93A*^*, Ncr1*^*gfp/gfp*^ and *SOD1*^*G93A*^*;Ncr1*^*gfp/gfp*^ mice (*n* = 3). (**F**) Representative flow cytometry plots from spinal cord single-cell suspensions of wildtype and *SOD1*^*G93A*^ mice at 140 days of age. Live CD45^+^ leukocytes were gated using Zombie NIR, then further resolved into NK1.1^+^ CD3^−^ and NK1.1^+^ CD3^+^ cell populations. Numbers indicate percentage of parent gate. (**G**,**H**) Frequency of NK1.1^+^CD3^−^ (G) and NK1.1^+^CD3^+^ (H) among CD45^+^ cells in the spinal cord of mice with indicated genotype. (**I**) Frequency of NK1.1^+^ cells identified from the CD45^int^CX3CR1^+^ resident microglial fraction (Suppl. Fig. 5A and B). Horizontal bars show mean values ± SD; *P < 0.05, n.s, non-significant; Kruskal-Wallis test with Dunn *post hoc* test (D,E) or Mann-Whitney test (G,H,I).

To not exclude the possibility that substantial changes of NK cells in the blood could occur at an earlier disease stage, we determined the frequency of populations expressing NK1.1, CD11b and KLRG1 at disease onset. We did not observe any significant change in the frequency of NK1.1^+^, CD11b^+^KLRG1^−^NK1.1^+^ and CD11b^+^KLRG1^+^NK1.1^+^ cell populations between wildtype and *SOD1*^*G93A*^ mice at 110 days of age (Suppl. Fig. 4A–C). Taken together, these results do not support a noticeable alteration in NK cell populations in the peripheral blood of ALS mice at 110, 140 and 150 days of age.

### 3.2 NK lymphocytes are not significantly infiltrating the CNS of SOD1^G93A^ mice

To investigate whether NK cells could be detected in the spinal cord of *SOD1*^*G93A*^ mice, we performed immunolabeling of GFP in spinal cord sections of *Ncr1*^*gfp/gfp*^ and *SOD1*^*G93A*^; *Ncr1*^*gfp/gfp*^ mice at 90 (presymptomatic), 110 (onset) and 140 (symptomatic) days of age. While GFP^+^ cells can be readily detected in spleen sections of *Ncr1*^*gfp/gfp*^, we did not observe any significant infiltration of GFP^+^ cells in the spinal cord of *Ncr1*^*gfp/gfp*^ and *SOD1*^*G93A*^; *Ncr1*^*gfp/gfp*^ at the presymptomatic, onset and progression stages of the disease (data not shown, Fig. 3A and B).

We analyzed by flow cytometry the CNS of 110-day-old *Ncr1*^*gfp/gfp*^ and *SOD1*^*G93A*^; *Ncr1*^*gfp/gfp*^ mice using CD45, CD3, Ncr1, NK1.1 and GFP (Fig. 3C). We first observed that the frequency of Ncr1^+^NK1.1^+^ in the CNS of *SOD1*^*G93A*^ mice at disease onset was not significantly different from that in age-matched wildtype mice (Fig. 3D). There was also no difference in GFP^+^NK1.1^+^ between *Ncr1*^*gfp/gfp*^ and *SOD1*^*G93A*^; *Ncr1*^*gfp/gfp*^ mice (Fig. 3E).

*Klrb1c*, which encodes the NK1.1 antigen, is a strongly upregulated transcript in spinal microglia of symptomatic *SOD1*^*G93A*^ mice (Chiot et al., 2020), and activated macrophages can themselves acquire NK1.1 surface expression in inflammatory settings (Steiger et al., 2015). We therefore adopted a complementary approach focusing on NK1.1 to assess whether its immunoreactivity in the ALS spinal cord may arise from cells of the myeloid lineage. We first analyzed the expression of CD45, CD3, and NK1.1 in spinal cord cells (Fig. 3F), and found that the proportion of CD45^+^CD3^−^NK1.1^+^ cells was not significantly different between wildtype and SOD1^G93A^ mice (Fig. 3G). However, we observed that the presumably natural killer T cells (NKT) cell population is significantly increased in the spinal cord of 140-day-old *SOD1*^*G93A*^ mice compared to their age-matched control mice (Fig. 3H), as previously described (Finkelstein et al., 2011). We then used CD45 and CX3CR1 to distinguish resident microglia (CD45^intermediate^CX3CR1^+^) from lymphoid cells (CD45^high^CX3CR1^-^) and CNS-associated macrophages (CD45^high^CX3CR1^+^)(Goldmann et al., 2016)(Suppl. Fig. 5A and B). Within the CD45^int^CX3CR1^+^ resident microglial population, the proportion of NK1.1^+^ cells were significantly increased in the spinal cord of *SOD1*^*G93A*^ mice compared to wildtype controls (Fig. 3I). We also observed that within the CD45^high^CX3CR1^-^ and CD45^high^CX3CR1^+^ cell populations, and among the CD3^−^ cells, expressing or not CD11b, a population of F4/80^+^ myeloid cells is significantly increased in *SOD1*^*G93A*^ mice (Suppl. Fig. 5C). This data shows that myeloid cells can express NK1.1 in the spinal cord of *SOD1*^*G93A*^ mice.

Our meta-analysis of CD49b^+^ within CD45^+^CD90.2^−^CD3^−^ cells in the CNS of wildtype and *SOD1*^*G93A*^ mice at 150 days of age (Coque et al., 2019), did not show any significant difference in the frequency of CD49b^+^ NK cells between the two groups (Suppl. Fig. 3C and D).

### 3.3 Loss of the NK cell-activating receptor Ncr1 in SOD1 mutant mice does not significantly influence motor decline or life span

We sought to determine whether the stimulatory receptor Ncr1 could have a function in the pathogenic events leading to motor deterioration. We explored the impact of the loss of Ncr1 function in SOD1 mutant mice on disease onset, defined as the timepoint when mice reach peak body weight, motor performance, assessed by grid test, and life expectancy (Coque et al., 2019). When we analyzed the time of onset, we did not observe any significant difference between *SOD1*^*G93A*^ *and SOD1*^*G93A*^ with a genetic deletion of *Ncr1* (*SOD1*^*G93A*^, 113.7 ± 2.9 d and *SOD1*^*G93A*^*; Ncr1*^*gfp/gfp*^ 121.8 ± 4.1 d, mean ± standard error of the mean (SEM), *n* = 15, *P* = 0.118, *t*-test)(Fig. 4A and B). We then investigated the impact of *Ncr1* deletion on motor decline of SOD1 mutant mice and found no statistical significance (Fig. 4C). We next determined the lifespan of *SOD1*^*G93A*^ and *SOD1*^*G93A*^*; Ncr1*^*gfp/gfp*^ mice, and found that the deletion of *Ncr1* neither reduced nor increased the mean lifespan of SOD1 mutant mice (*SOD1*^*G93A*^, 166.9 ± 2.1 and *SOD1*^*G93A*^*; Ncr1*^*gfp/gfp*^, 163.9 ± 2.7, mean ± SEM, *n* = 15, *P* = 0.566, Log-rank Mantel-Cox test)(Fig. 4D). Administration of an NK1.1-depleting antibody in *SOD1*^*G93A*^ mice was shown to increase survival in female, but not male, mice (Murdock et al., 2021). We then analyzed onset and lifespan separately by sex and found no significant difference between males and females in either *SOD1*^*G93A*^ and *SOD1*^*G93A*^*; Ncr1*^*gfp/gfp*^ cohorts (Suppl. Fig. 6A and B).

**Fig. 4:**
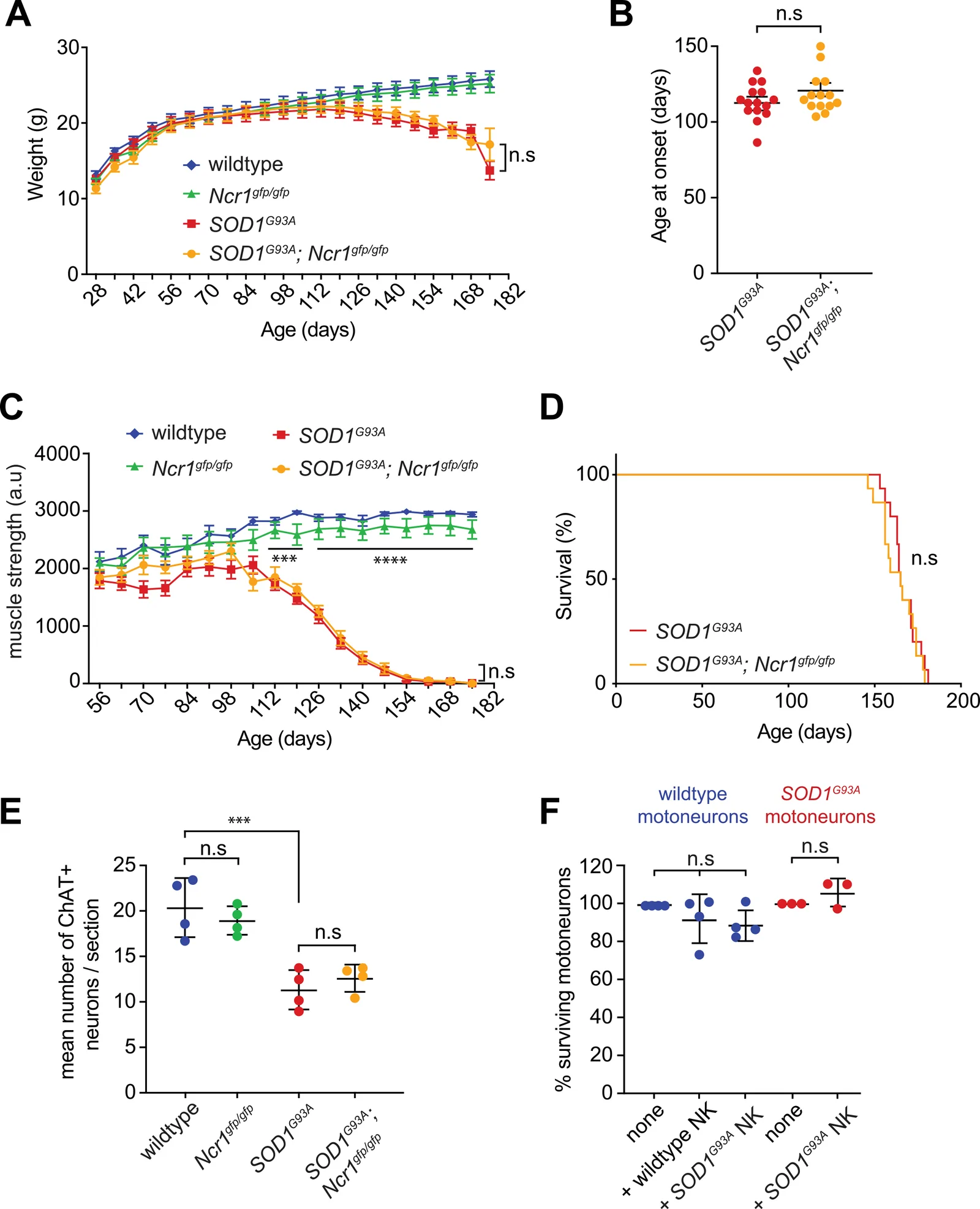
***Ncr1* deletion does not alter disease course or motoneuron survival in *SOD1*^*G93A*^ mice** (**A**) Body weight curves of wildtype, *SOD1*^*G93A*^*, Ncr1*^*gfp/gfp*^ and *SOD1*^*G93A*^*; Ncr1*^*gfp/gfp*^ mice (*n* = 15 per genotype, mixed sex groups). Two-way ANOVA with Newman-Keuls *post hoc* test, n.s, non-significant. (**B**) Age of disease onset of *SOD1*^*G93A*^*; Ncr1*^*+/+*^ and *SOD1*^*G93A*^*; Ncr1*^*gfp/gfp*^ mice. Disease onset was defined as the age at which mice reached peak body weight curve (*n* = 15), Values are means ± SEM; unpaired two-tailed *t*-test. (**C**) Muscle strength of wildtype, *SOD1*^*G93A*^*, Ncr1*^*gfp/gfp*^ and *SOD1*^*G93A*^*; Ncr1*^*gfp/gfp*^ mice was assessed weekly starting at 56 days of age by grid test. Values are means ± SEM from 15 mice; 2-way ANOVA with Newman-Keuls *post hoc* test. Statistical attributes are shown for *Ncr1*^*gfp/gfp*^ versus *SOD1*^*G93A*^*; Ncr1*^*gfp/gfp*^ groups, ***P < 0.001 and ****P < 0,0001. (**D**) Kaplan-Meier survival curve of *SOD1*^*G93A*^ and *SOD1*^*G93A*^*; Ncr1*^*gfp/gfp*^ mice (*n* = 15). Log-rank Mantel-Cox test. (**E**) The number of ChAT^+^ motoneurons was determined in spinal cord sections of mice of indicated genotype at 140 days of age (*n* = 4). Values are expressed as mean ± SD; one-way ANOVA with Tukey's *post hoc* test. (**F**) Wildtype and *SOD1*^*G93A*^ motoneurons were seeded (or not, none) with NK cells isolated from symptomatic mice of the indicated genotype (with a ratio of 1 motoneuron for 10 NK cells). Motoneuron survival was determined 48 h later and expressed relative to the survival in the absence of NK cells. Values are means ± SEM from 3 to 4 independent experiments; one-way ANOVA with Tukey-Kramer *post hoc* test.

Motoneuron survival was then assessed by determining the number of choline acetyltransferase (ChAT)-positive motoneurons in the spinal cord of 140-day-old symptomatic *SOD1*^*G93A*^ and *SOD1*^*G93A*^*; Ncr1*^*gfp/gfp*^ mice and age-matched wildtype and *Ncr1*^*gfp/gfp*^ control mice. We did not observe a significant difference in the number of spinal motoneurons between *SOD1*^*G93A*^ and *SOD1*^*G93A*^*; Ncr1*^*gfp/gfp*^ mice, while, as expected, expression of SOD1 mutant alone led to a marked loss of motoneurons compared to wildtype and *Ncr1*^*gfp/gfp*^ (Fig. 4E). These results show that the loss of the cytotoxicity-activating receptor Ncr1 function does not have functional consequences on the ALS-related deterioration of the motor system.

We then sought to evaluate the potential neurotoxicity of NK cells toward motoneurons *in vitro*, as it was suggested that SOD1^G93A^-expressing NK cells could show cytotoxicity against motoneurons through the activating receptor NKG2D (Garofalo et al., 2020). Embryonic motoneurons from wildtype and *SOD1*^*G93A*^ mice were co-cultured with NK cells from 130-day-old wildtype or *SOD1*^*G93A*^ mice. The percentage of surviving motoneurons was not significantly changed by the presence of NK cells after 48 h of culture (Fig. 4F).

### 3.4 The Ncr1 ligand, properdin, labels misfolded SOD1 inclusions and degenerating astrocytes

To validate our observation, we focused our attention on properdin, which can trigger non-canonical NK cell activation (Narni-Mancinelli et al., 2017), and also tags dying cells, helping phagocytes to eliminate damaged cells (Kemper et al., 2008; Xu et al., 2008). Following ischemic stroke, Properdin levels increase in the brain, which is produced by infiltrating myeloid cells, and promotes inflammation by activating microglial cells (Liu et al., 2023). We observed a significant accumulation of Properdin in the spinal cord of symptomatic *SOD1*^*G93A*^ compared to wildtype and *SOD1*^*G93A*^ mice at disease onset (Fig. 5A and B). To validate this staining, a second antibody against Properdin was used, yielding comparable staining patterns (Suppl. Fig. 7A). Properdin is a soluble pattern recognition molecule that binds to DNA exposed by dying cells or functionalized nanoparticles (Kouser et al., 2018; Xu et al., 2008). We first asked whether Properdin has the capacity to recognize SOD1 aggregates that are able to bind to pattern recognition receptors (Gosset et al., 2022). We used the B8H10 misfolded SOD1-specific monoclonal antibody to identify the deposits of misfolded SOD1 in the spinal cord of symptomatic SOD1 mutant mice (Gros-Louis et al., 2010). We observed that Properdin decorated misfolded SOD1 deposits (Fig. 5C). Properdin directly activating microglia cells through Mincle receptor (Liu et al., 2023), we performed co-immunostaining for misfolded SOD1, Properdin and CD45, the latter being expressed at high levels by microglia. We found that the misfolded SOD1 structures labeled by Properdin could be found associated with CD45^+^ cells with large soma and limited ramification, reminiscent of reactive microglia (Savage et al., 2019)(Fig. 5D). Confocal microscopy with 3D reconstruction shows that CD45^+^ cells closely wrap around the Properdin-tagged structures within the spinal cord of 150-day-old *SOD1*^*G93A*^ mice (Fig. 5E). CD68, which promotes phagocytosis is found surrounding Properdin-decorated elements in the *SOD1*^*G93A*^ spinal cord (Fig. 5F). The colocalization of CD68 with Iba1 (Fig. 5G), suggests that Properdin may act as a phagocytic signal. Together, the combination of reactive microglia-like morphology (large soma with limited ramification), Iba1 expression, and CD68-associated phagocytic activity supports the interpretation that Properdin-associated cells correspond to activated, phagocytic microglia rather than infiltrating leukocytes.

**Fig. 5:**
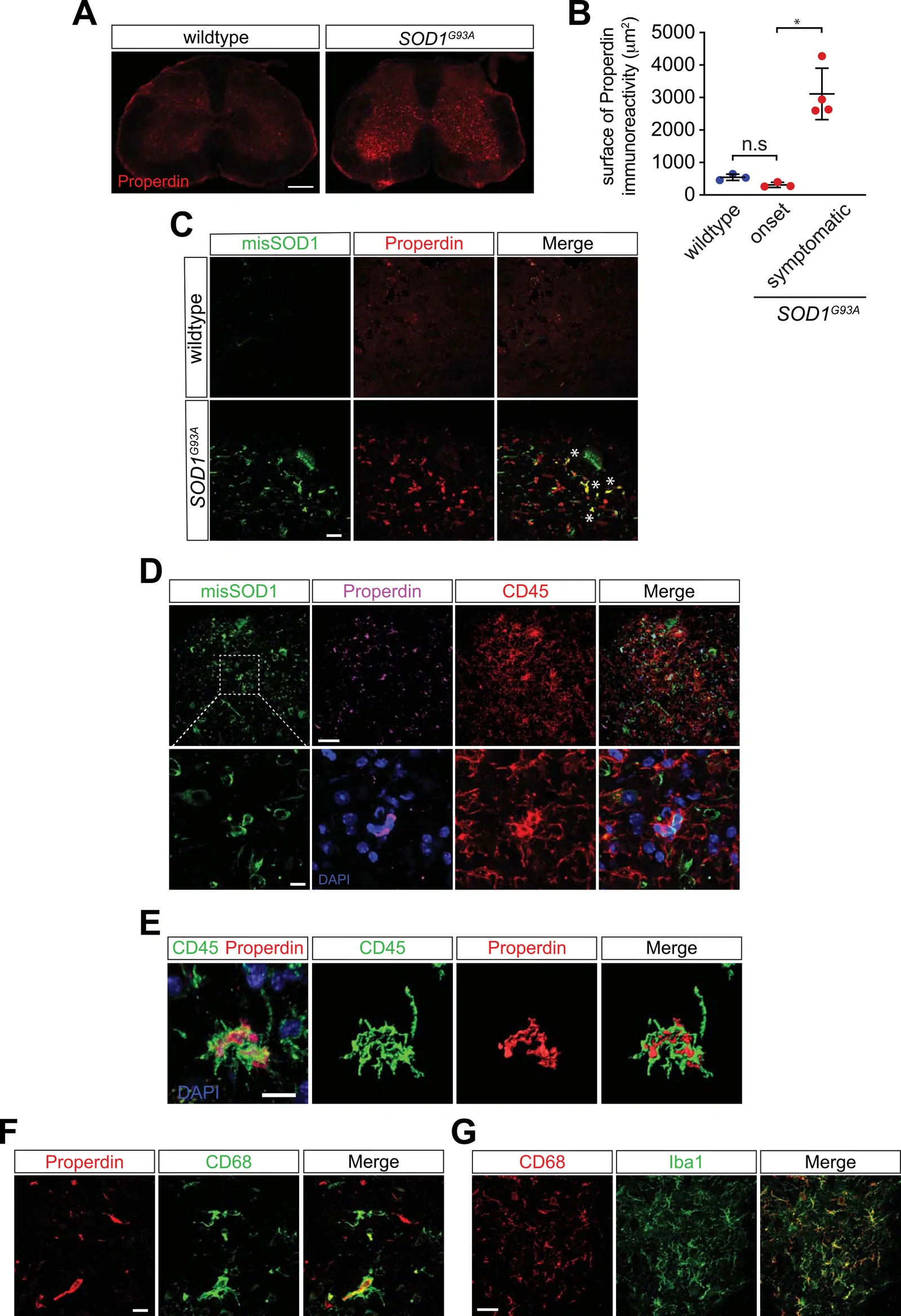
**Properdin accumulates in the spinal cord of *SOD1*^*G93A*^ mice and may act as a phagocytic signal for microglia**. (**A**) Lumbar spinal cord sections from wildtype and *SOD1*^*G93A*^ at 150 days of age were immunostained with anti-Properdin antibodies. Scale, 50 μm. (**B**) Quantification of the total Properdin immunostained surface in the ventral horn of wildtype (150 days, *n* = 3), *SOD1*^*G93A*^ at onset (110 days, *n* = 3) and symptomatic stage (150 days, *n* = 4). Values are means ± SD; n.s, non-significant, *P < 0.01; Kruskal-Wallis test with Dunn *post hoc* test. (**C**) Spinal cord sections from 150-day-old wildtype and *SOD1*^*G93A*^ mice were immunostained with antibodies against the misfolded SOD1 (green) and Properdin antibody (red). Scale 25 μm. Stars indicate the overlay of two immunostaining signals. (**D**) Representative image of the ventral horn of symptomatic-stage ALS mice stained with antibodies against misfolded SOD1 (green), Properdin (magenta) and CD45 (red). Scale bar, 50 μm. The area boxed in the upper panel is presented at higher magnification in the lower panels. DAPI is in blue. Scale bar 10 μm. (**E**) Confocal and 3D reconstructed images showing wrapping around Properdin structures by CD45^+^ cells in the spinal cord of *SOD1*^*G93A*^ mice. Scale bar, 10 μm. (**F**) Representative image of spinal cord section immunolabeled with Properdin (red) and the lysosomal marker CD68 (green). Scale bar, 10 μm. (**G**) Co-labeling of CD68 (red) with the microglial cell marker Iba1 (green). Scale bar, 50 μm.

The size of some structures labeled by Properdin (around 10 μm, Fig. 5D–F) and the presence of DAPI staining suggest that Properdin marks cells undergoing cell death, consistent with its role in opsonizing apoptotic or necrotic cells (Kemper et al., 2008; Xu et al., 2008). We first asked whether Properdin label motoneurons, but found no co-labeling with ChAT (Fig. 6A). Since aberrant degenerating astrocytes accompany motoneuron loss in ALS (Crabe et al., 2020; Rossi et al., 2008; Trias et al., 2013), we next examined whether these cells could be targeted by Properdin. We identified Properdin-labeled cells displaying the hallmark of degenerating astrocytes: a spheroid shape, blurred somatic GFAP staining and dense GFAP staining in shortened proximal process (Fig. 6B). These cells also expressed cleaved caspase-3 (Fig. 6C) and ubiquitin (Fig. 6D), two established markers of degenerating astrocytes in ALS (Rossi et al., 2008).

**Fig. 6:**
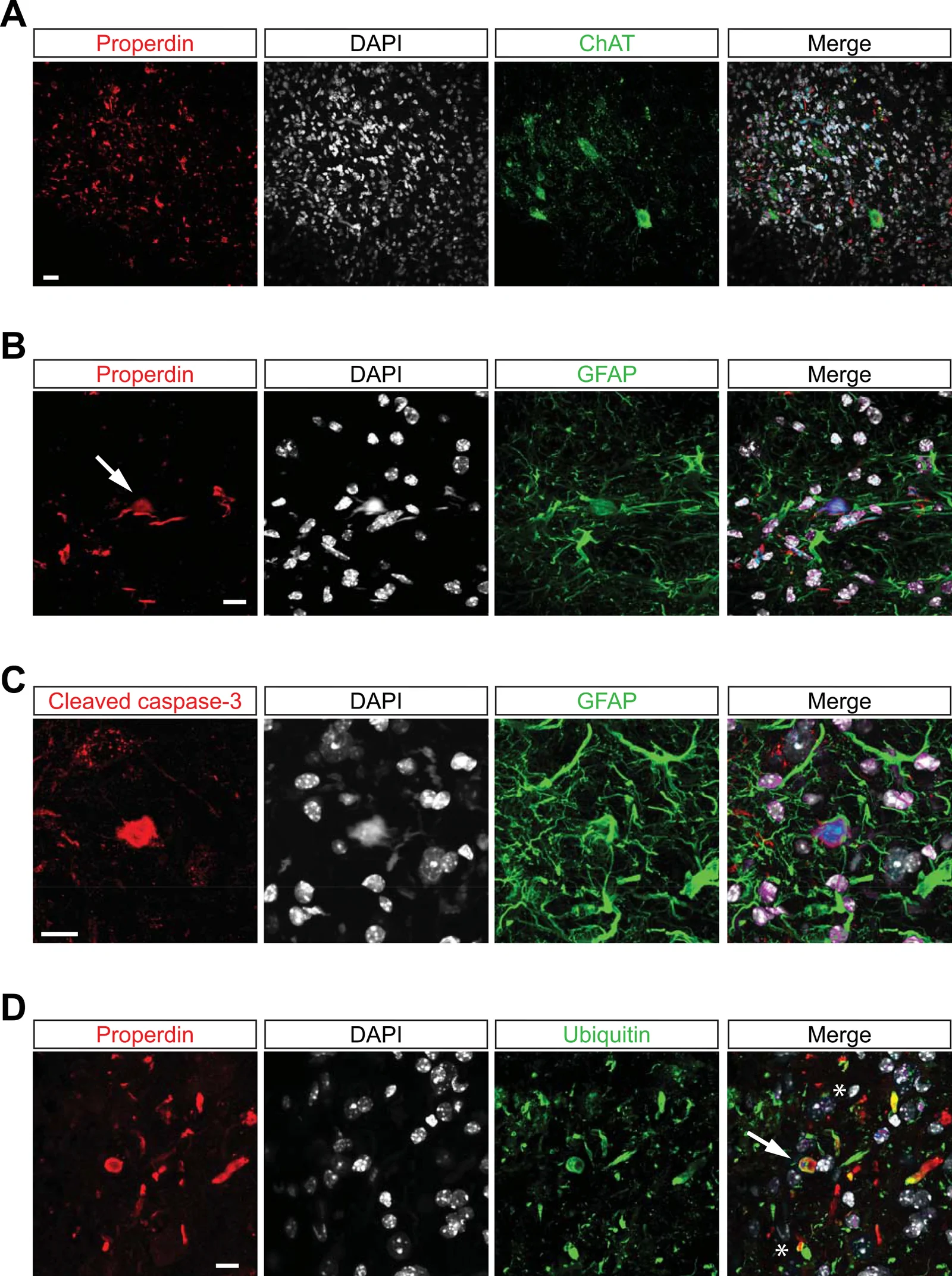
**Properdin localizes to degenerating astrocytes in the spinal cord of *SOD1*^*G93A*^ mice** (**A**) Representative image showing the localization of ChAT^+^ motoneurons (green) and Properdin (red) in the spinal cord of 150-day-old SOD1^G93A^ mice. DAPI in white. Scale 20 μm. (**B**) Confocal image showing spheroid-shaped Properdin cell body (in red, white arrow) immunolabeled with GFAP (green). Scale bar, 10 μm. (**C**) Co-labeling of spheroid shape GFAP^+^ cells (green) with antibodies against the cleaved form of caspase-3 (red) in the spinal cord of ALS mutant mice at symptomatic stage. DAPI in white. Scale bar, 10 μm. (**D**) Representative image of spinal cord section immunostained with antibodies directed against Properdin (red) and ubiquitin (green). The white arrow indicates the spheroid-shaped cell body. Scale bar, 10 μm.

### 3.5 Targeted deletion of Properdin in SOD1^G93A^ mice

To investigate the role of Properdin in ALS mice, we crossed *SOD1*^*G93A*^ mice with mice a target deletion of exons coding for the thrombospondin repeats, which are important for Properdin oligomerization (Stover et al., 2008). *Properdin*^*−/−*^ mice show no gross abnormalities, are fertile and do not exhibit weight loss compared to their wildtype littermate. However, *Properdin*^*−/−*^ mice show increased susceptibility to bacterial pathogens, abolition of terminal complement activation and a skewing of macrophages toward M2 phenotype (Al-Rayahi et al., 2017; Jain et al., 2015; Stover et al., 2008). We analyzed the impact of Properdin loss of function on the ALS-associated neuroinflammatory response and found that the number of CD45^+^-positive cells, although significantly increased at the symptomatic stage in *SOD1*^*G93A*^ mice, was not changed in the spinal cord of *SOD1*^*G93A*^*; Properdin*^*−/−*^ mice (Fig. 7A). A significant increase in GFAP^+^ cells was observed in 150-day-old SOD1 mutant compared to wildtype mice. However, the targeted deletion of *Properdin* did not alter the number of astrocytes in the ventral horn of the spinal cord compared to *SOD1*^*G93A*^ mice (Fig. 7B). Furthermore, the number of spinal GFAP^+^/cleaved caspase-3^+^ degenerative astrocytes was not affected by *Properdin* deletion in SOD1 mutant mice (Fig. 7C). We then analyzed whether the deletion of *Properdin* could have an impact on the loss of motoneurons. While a significant decrease of spinal motoneuron survival is observed in *SOD1*^*G93A*^ mice at the symptomatic stage, we did not find any difference between *SOD1*^*G93A*^ and *SOD1*^*G93A*^*; Properdin*^*−/−*^ (Fig. 7D and E). Together, our observations do not suggest a significant role of Properdin in the neuroinflammatory response and motoneuron survival in ALS mice.

**Fig. 7:**
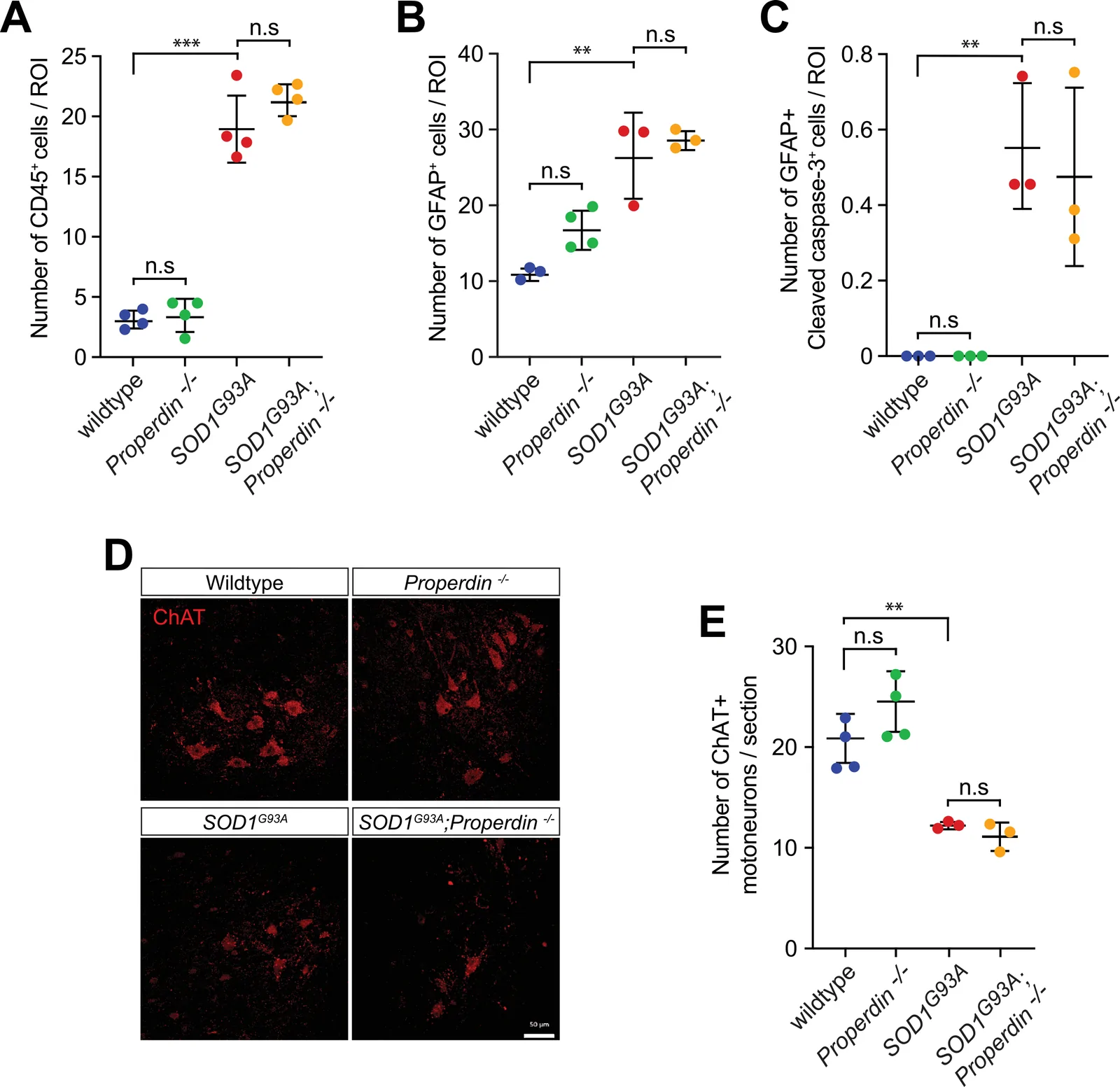
***Properdin* deletion does not prevent neuroinflammation or motoneuron loss in *SOD1*^*G93A*^ mice**. (**A**-**C**) Lumbar spinal cord sections from 150-day-old wildtype, *Properdin*^*−/−*^*, SOD1*^*G93A*^ and *SOD1*^*G93A*^*; Properdin*^*−/−*^ mice were immunostained with antibodies against CD45, GFAP and cleaved caspase-3. The number of CD45^+^ (A), GFAP^+^ (B) and double-positive GFAP and cleaved caspase-3 (C) cells was counted in a region of interest (ROI), defined as a disc in the ventral grey matter of the spinal cord with a standard area of 4823.6 μm^2^. (**D,E**) Spinal cord sections from wildtype (*n* = 4), *Properdin*^*−/−*^ (*n* = 4), *SOD1*^*G93A*^ (*n* = 3) and *SOD1*^*G93A*^*; Properdin*^*−/−*^ (*n* = 3) mice at 150 days of age were stained with anti-ChAT antibodies, and the number of surviving motoneurons was determined. Values are mean ± SD, one-way ANOVA with Tukey-Kramer *post hoc* test, n.s, non-significant, **P < 0.01 and ***P < 0.001.

## 4 Discussion

In this study, we combined comprehensive immunophenotyping with genetic loss-of-function approaches targeting *Ncr1* and *Properdin* to trace NK cells and evaluate their Ncr1-dependent contribution to ALS pathogenesis. We found no change in the number, maturation, or activation status of circulating NK cells in *SOD1*^*G93A*^ mice compared to wildtype littermates. Contrary to previous reports (Chiu et al., 2008; Garofalo et al., 2020; Komine et al., 2018), we did not detect significant NK cell infiltration in the CNS of *SOD1*^*G93A*^ mice, nor did SOD1^G93A^-expressing NK cells affect motoneuron survival *in vitro*. Genetic ablation of *Ncr1* did not influence disease onset, motor decline, motoneuron survival, or lifespan. Properdin decorated misfolded SOD1 aggregates and degenerating astrocytes, consistent with a role as a phagocytic signal for microglia, yet its targeted ablation did not influence neuroinflammation or motoneuron survival. Together, these convergent findings do not support a substantial contribution of NK cells to disease progression in ALS mice.

### 4.1 Ncr1 (NKp46)-expressing innate lymphoid cells

In human, NKp46/NCR1 was initially described as a NK-specific receptor involved in NK activation triggering non-MHC-restricted cytotoxicity (Pessino et al., 1998). Since then, NKp46/NCR1 has been found to be expressed by innate lymphoid cells (ILC), by Vδ1^+^ T cells, a discrete subset of T lymphocytes with cytolytic properties against some hematologic tumor cells (Correia et al., 2011), as well as by a subset of gut intraepithelial lymphocytes (Ettersperger et al., 2016).

ILCs represent a heterogeneous group of immune cells that encompass NK cells, group 1 (ILC1), group 2, (ILC2), and group 3 (ILC3). ILC3s are further subdivided into lymphoid tissue-inducer-like ILC3s and Ncr1^+^ ILC3s (van de Pavert and Vivier, 2016). Despite the expression of the NK cytotoxic receptor, Ncr1^+^ ILC3s are more commonly described for their ability to promote inflammation than for their lytic functions. ILC3s are commonly retrieved in mucosal surfaces, where they play an important role in maintaining mucosal tissue homeostasis, as well as in secondary lymphoid organs, meninges and the CNS (Brown and Weinberg, 2018). In experimental autoimmune encephalomyelitis, a murine model of multiple sclerosis, Ncr1^+^ ILC3s accumulate in the meninges, brain and spinal cord (Hatfield and Brown, 2015), where they regulate neuroinflammation by controlling the infiltration and stability of CD4^+^ Th17 cells (Kwong et al., 2017).

Relevant to ALS, NKp46^+^ immunolabeled cells have been observed in the CNS of ALS patients and *SOD1*^*G93A*^ mice by immunohistochemistry (Garofalo et al., 2020). In contrast, the present study failed to detect any GFP^+^ cells in the spinal cord parenchyma of *SOD1*^*G93A*^ mice using *Ncr1*^*gfp/gfp*^ as reporter line. Several methodological factors may account for this discrepancy. First, GFP expression driven by the endogenous Ncr1 promoter may fall below the detection threshold of immunohistochemistry, particularly if Ncr1 transcriptional activity is low or downregulated in the CNS microenvironment. Second, NK cells and ILC3s are known to modulate surface receptor expression upon tissue entry, and loss of Ncr1 expression within the CNS cannot be excluded. Third, and critically, the meningeal compartment, where ILC3s preferentially reside, requires specialized tissue processing approaches, including non-conventional spinal cord extrusion, longitudinal sectioning, or whole-mount preparations (DiSano et al., 2020; Kwong et al., 2017; Rua et al., 2019), none of which were employed here. It is therefore possible that Ncr1^+^ ILCs are present in the spinal cord meninges but were missed by conventional coronal sectioning of the parenchyma. Future studies using these dedicated approaches will be necessary to determine whether and how ILC3s contribute to the immune response in ALS. Beyond ILC3s, a distinct Ncr1-expressing ILC subset -intraepithelial and lamina propria ILC1s-has been implicated in inflammatory bowel disease (Fuchs et al., 2013). Our study suggests that Ncr1-dependent functions, both in NK cells and in the other Ncr1-expressing immune cell populations, do not appear to play a substantial role in ALS. This does not exclude, however, that ILCs or Vδ1^+^ T cells populations may contribute to ALS through Ncr1-independent mechanisms.

### 4.2 NK1.1 in spinal microglia, activated macrophages and innate lymphoid cells

NK1.1 cell surface expression in CD45^+^CD3^−^ cells has been used to quantify the infiltration of NK cells in the CNS of ALS mice. Interestingly, analysis of the spinal microglial transcriptome of *SOD1*^*G93A*^ mice identified *Killer cell lectin-like receptor subfamily B member 1c* (*Klrb1c*) as the most upregulated gene between onset/early symptomatic and presymptomatic stages of the disease (Chiot et al., 2020). Consistently, we observed that resident microglial cells acquire the NK1.1 in the spinal cord of symptomatic *SOD1*^*G93A*^ mice (Suppl. Fig. 4).

NK1.1 can also be found expressed at the surface of other CD45^+^ hematopoietic cells that are negative for the prototypic marker of T lymphocytes, CD3. Indeed, in response to some specific inflammatory signals, macrophages exhibit surface expression of NK1.1 and exert a perforin-dependent cytotoxic activity toward target cells (Steiger et al., 2015). The commonly used anti-NK1.1 (clone PK136) blocking/depleting antibody significantly decreases the killing activity of NK1.1^+^ macrophages (Steiger et al., 2015). It has also been demonstrated that lipopolysaccharide-activated macrophages can express NKG2D, whose activation by H-60 or Rae1β NKG2D ligands promotes macrophage effector functions, such as the production of nitric oxide and tumor necrosis factor alpha (TNFα)(Diefenbach et al., 2000). Macrophages from naked mole-rats were also characterized by the expression of CD11b and NK1.1. Activation of NK1.1 in these macrophages leads to morphological changes as well as their proliferation (Wada et al., 2019). It is noteworthy that a population of macrophages can acquire effector features and can directly kill target cells through the NKG2D and perforin/granzyme B pathway (Baba et al., 2008; Chu et al., 2020).

In the liver at physiological conditions, about 60% of the CD45^+^CD3^−^NK1.1^+^ cells are classic CD49b^+^ NK cells and 40% are resident ILC1 (Chen et al., 2017). Importantly, ILC1 can also be observed in the CNS (Romero-Suarez et al., 2019), where they can be distinguished from NK cells by the expression of CD49a and CD49b, as well as other markers, albeit less stringent (CD200R, CXCR6, TRAIL and DNAM-1). The phenotypic profile of ILC1 in the CNS being CD45^high^CD3^−^NK1.1^+^CD49a^+^CD49b^−^. ILC1 of the CNS express Perforin and Granzyme B and upon stimulation produce TNFα and IFNγ. During CNS inflammation, the number of ILC1 (as well as intermediate ILC1) increases in the parenchyma of the brain, and might derive from infiltration from other meningeal compartments (Romero-Suarez et al., 2019). In the present study, we carefully combined a series of lymphoid and myeloid markers to narrow any overlap with cell types other than NK. However, we were unable to demonstrate either significant NK infiltration into the CNS or variation in the number of NK cells in the circulating blood of ALS mice. However, it remains of interest to explore the relative contribution of NK1.1^+^ macrophages and ILC1 populations in the pathogenesis of the disease.

### 4.3 Peripheral disturbance of NK cells in ALS

Our immunophenotyping of circulating NK cells showed that neither their representation, maturation nor activation status differed between ALS and control mice harboring or not a targeted deletion of the *Ncr1* locus. In agreement, a previous report showed that the percentage of NK cells in the spleen and liver did not differ between wildtype and *SOD1*^*G93A*^ mice (Finkelstein et al., 2011). Studies that combined the surface expression of phenotypic markers CD3, CD16 and CD56 observed a significant increase of NK cells in the circulating blood of patients with ALS compared to healthy subjects (Gustafson et al., 2017; Jin et al., 2020; Murdock et al., 2017; Rentzos et al., 2012). This increase is in conjunction with a peripheral inflammatory response as defined by the pro-inflammatory feature of innate and adaptive immune cells and the impressive panel of cytokines which are found elevated in the blood and cerebrospinal fluid of ALS patients (McCombe et al., 2020; Moreno-Martinez et al., 2019). However, data reported so far include healthy controls but not patients with chronic inflammatory disease or other neurological disorders with or without peripheral inflammatory components. Additional studies including comprehensive cohorts could identify an ALS-specific immune signature in cell subpopulation and cytokine profiles. This can be of particular importance knowing the more global function of NK cells in immune surveillance or disease control, as illustrated in the resolution of acute allergic airway inflammation or the diminution of local inflammatory response in the CNS (Hao et al., 2010; Haworth et al., 2011). Levels of IL-12, IL-18 and IL-15, which stimulate NK proliferation and activation, are found elevated in blood of ALS patients compared to controls (Moreno-Martinez et al., 2019). This is of particular interest knowing that these cytokines can independently of target cells, restimulate a population of memory-like NK cells, which have been previously activated by tumor cells or following virus infection, to produce IFNγ with an inherited capacity to proliferate (Pahl et al., 2018; Romee et al., 2012).

However, another study using CD3 and CD56 as discriminating determinants observed a reduced frequency of NK cells in peripheral blood (Garofalo et al., 2020). Differences in clinical characteristics, cohort size, and methodological factors might be considered to explain the variability among studies. Moreover, the standardization of immunophenotypic analysis is cardinal, since for instance some populations of activated plasmacytoid, IL-15-induced myeloid dendritic cells, ILC-1-like cells and NKT cells express CD56 (Anguille et al., 2012; Salome et al., 2019; Tel et al., 2012).

Recently, a single cell RNA sequencing analysis of human ALS brain and spinal cord CD45^+^ immune cells show an enrichment of NK cells and maturing NK cells in ALS samples (Tuddenham et al., 2025). These findings highlight the significant difficulty in obtaining a clear, unified understanding of NK cell dynamics across the CNS and circulating blood in humans.

### 4.4 Properdin

Properdin is primarily a positive regulator of the alternative complement pathway and acts as a pattern recognition molecule, identifying and binding to dying cells to facilitate their removal by complement activation and phagocytosis (Kemper et al., 2008; Xu et al., 2008). In the brain, during an ischemic stroke, Properdin, whose levels are elevated, promotes activation of microglia and thus enhances the production of inflammatory cytokines. Stimulation of microglial cells by recombinant Properdin leads to their activation, without leading to an upregulation of CD68, but causes cortical neuron death *in vitro* associated with increased expression levels of IL-1β and TNFα (Liu et al., 2023). We observe, in the context of ALS, that Properdin is also capable of recognizing a surface such as inclusions of misfolded SOD1 or degenerating astrocytes. Properdin can then be recognized by phagocytic CD68^+^ microglia in the spinal cord, likely acting as an opsonin. On the other hand, the absence of Properdin, contrary to what is observed in ischemic stroke with brain neurons (Liu et al., 2023), does not increase the number of surviving motoneurons in the spinal cord of ALS mice. Of note, this endpoint was assessed in a slightly smaller cohort (*n* = 3, Fig. 7E); nevertheless, this result is consistent with the unchanged neuroinflammatory profile observed in the same cohort (Fig. 7A and B). Our observation that the loss of Properdin function does not change the neuroinflammatory response or affect motoneuron survival is interestingly paralleled by observations made with another component of the complement system, C1q. C1q that activates the classical complement pathway after binding to different surfaces, including dying cells or beta-amyloid and promote phagocytosis (Parker et al., 2019). Deletion of C1q in *SOD1*^*G37R*^ mice does not impact the neuroinflammatory response or motoneuron loss (Lobsiger et al., 2013). In addition, loss of C3, a central component downstream of both the alternative and classical pathways, does not change the course of the disease and motoneuron degeneration in SOD1 mutant mice (Lobsiger et al., 2013). Although the inactivation of these upstream components of the complement does not appear to play a significant role in degeneration and the neuroinflammatory status, it does not necessarily exclude the involvement of the complement system in the pathogenesis of ALS, as illustrated by the terminal component C5, whose inhibition results in an increased lifespan and improved motor functions in ALS mice (Lee et al., 2017; Woodruff et al., 2014). Our observations on the deletion of Properdin, as a ligand of NCR1, are consistent with the absence of effect of Ncr1 deficiency on the development and progression of the pathology.

## 5 Conclusion

Our results provide new insights into the potential involvement of NK cells in ALS pathogenesis. While previous studies have suggested a role for NK cells in the disease, including reports of their infiltration into the CNS and possible cytotoxic effects, our convergent findings argue against a major pathogenic role of these cells in the *SOD1*^*G93A*^ mouse model. Specifically, we observed no significant differences in NK cell number, maturation, or activation between ALS and control mice, nor did we detect notable NK cell infiltration in the CNS; together with the absence of effect of *Ncr1* genetic ablation on disease onset, motor function decline, or survival, these convergent findings argue against a major pathogenic role of NK cells in the *SOD1*^*G93A*^ mouse model. Although Properdin, a ligand of NKp46/Ncr1, was found to associate with misfolded SOD1 aggregates and degenerating astrocytes, its deletion similarly had no impact on neuroinflammation or motoneuron survival. Nonetheless, our findings are limited to a single transgenic mouse model and do not address the meningeal compartment or ILC subsets beyond NK cells. Further studies in human patients and complementary model systems may be needed to clarify the relevance of NK cells and other ILCs in ALS pathogenesis.

## CRediT authorship contribution statement

**Roxane Crabé:** Writing – original draft, Methodology, Investigation, Formal analysis. **Alexandre Brodovitch:** Writing – original draft, Methodology, Investigation, Formal analysis. **Philippe Gosset:** Investigation, Formal analysis. **Charlotte Gavard:** Investigation, Formal analysis. **Véronique Brugioti:** Investigation. **Désiré Challuau:** Investigation. **Antoine Picot:** Investigation. **Gabriel Espinosa-Carrasco:** Investigation, Formal analysis. **Laurent Soustelle:** Investigation, Formal analysis. **Kevin Mouzat:** Formal analysis. **Javier Hernandez:** Formal analysis. **José Boucraut:** Formal analysis. **Thierry Vincent:** Formal analysis. **Cédric Raoul:** Writing – review & editing, Writing – original draft, Validation, Supervision, Project administration, Investigation, Funding acquisition, Formal analysis, Conceptualization.

## Ethics declaration

This study was conducted in accordance with the ARRIVE (Animal Research: Reporting of In Vivo Experiments) guidelines. This study was approved by the Ethics Committee for Animal Experimentation - Languedoc Roussillon (CEEA-LR).

## Funding

This work was supported by grants from the national institute for health and medical research (Inserm), the association pour la recherche sur la SLA (ARSLA) and the fondation pour la recherche médicale (FRM). R.C. was a recipient of a ministère de l'enseignement supérieur, de la recherche et de lʼinnovation (MESRI) and ARSLA Ph.D fellowship. P.G was a recipient of a French national research agency (ANR SPREADALS) and ARSLA Ph.D fellowship, C.G is a recipient of ARSLA Ph.D fellowship.

## Declaration of competing interest

The authors declare that they have no known competing financial interests or personal relationships that could have appeared to influence the work reported in this paper.

## Figures and Tables

**Figure:**
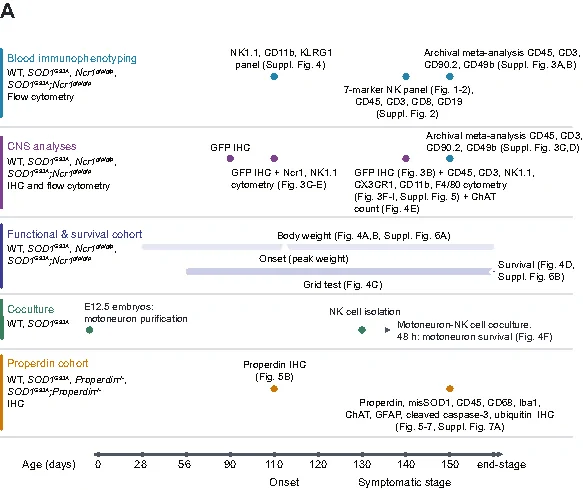
Supplementary Figure 1 legend. Schematic overview of the experimental design and timeline. Age of the mice (in days) is indicated on the horizontal axis (intervals not to linear scale). Cohorts, procedures performed and corresponding figure(s) are indicated.

**Figure:**
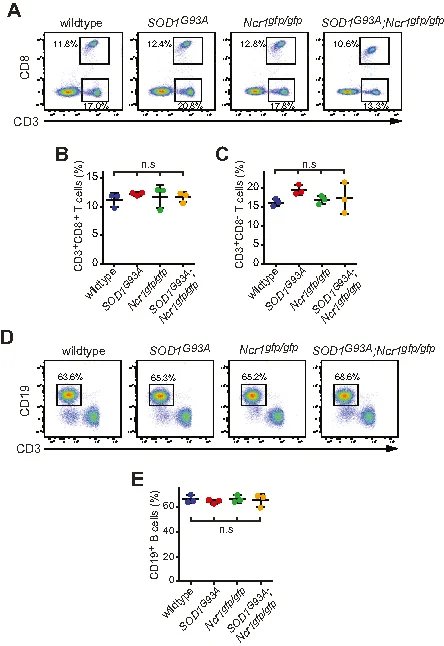
Supplementary Figure 2 legend (**A**) Representative flow cytometry plots of CD3^+^CD8^+^ and CD3^+^CD8^−^ T cells from peripheral blood of wildtype, *SOD1*^*G93A*^, *Ncr1*^*gfp/gfp*^ and *SOD1*^*G93A*^; *Ncr1*^*gfp/gfp*^ mice at 140 days of age. Numbers indicate percentage of gated populations. (**B**,**C**) Quantification of CD3^+^CD8^+^ T cells (B) and CD3^+^CD8^−^ T cells (C) as a percentage of circulating leukocytes. (**D**) Representative flow cytometry plots of CD19^+^CD3^−^ B cells across the four genotypes. (**E**) Quantification of CD19^+^ B cells as a percentage of total leukocytes. Data are means ± SD; n.s, non-significant; Kruskal-Wallis test with Dunn *post hoc* test.

**Figure:**
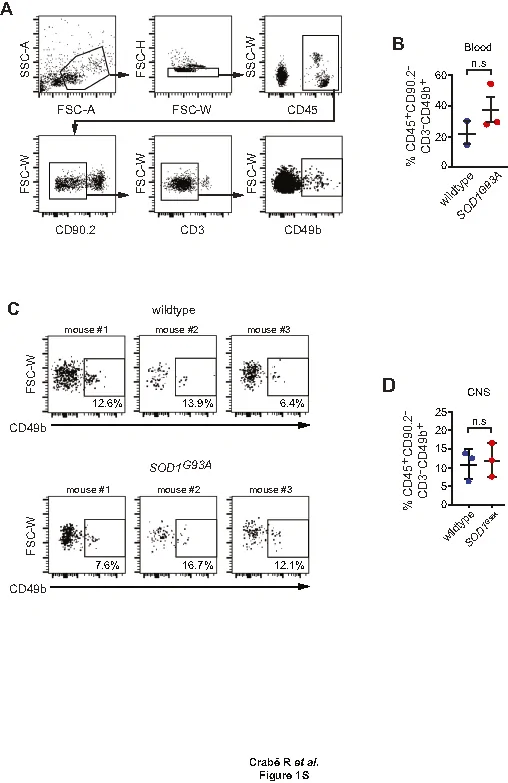
Supplementary Figure 3 legend. (**A**) Gating strategy used to identify NK cells in peripheral blood. Leukocytes were first gated based on forward scatter area/side scatter area (FSC-A/SSC-A), then on singlets (forward scatter height/width, FSC-H/FSC-W), CD45^+^ cells, then CD90.2^−^CD3^−^CD49b^+^ cells to define the NK cell population. (**B**) Quantification of CD45^+^CD90.2^−^CD3^−^CD49b^+^ NK cells as a percentage of CD45^+^ cells in peripheral blood of 150-day-old wildtype and *SOD1*^*G93A*^ mice. Data are means ± SD; n.s., not significant; Mann-Whitney test. (**C**) Application of the same gating strategy to spinal cord and brain cells. Each plot represents an individual mouse showing the final CD49b gate within the CD45^+^CD90.2^−^CD3^−^ population. Numbers indicate percentage of CD49b^+^ NK cells. (**D**) Percentage of CD45^+^CD90.2^−^CD3^−^CD49b^+^ NK cells in the CNS of wildtype and *SOD1*^*G93A*^ mice at 150 days of age. Values are means ± SD; n.s, non-significant; Mann-Whitney test.

**Figure:**
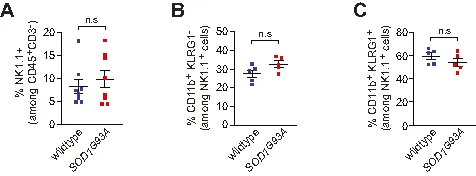
Supplementary Figure 4 legend (**A**) Percentage of NK1.1^+^ cells among CD45^+^CD3^−^ leukocytes in peripheral blood from wildtype and *SOD1*^*G93A*^ mice at 110 days of age. (**B**,**C**) Frequency of CD11b^+^KLRG1^-^ (B) and CD11b^+^KLRG1^+^ (C) mature NK cells among NK1.1^+^ cells. Data are means ± SD; n.s., not significant; Mann-Whitney test.

**Figure:**
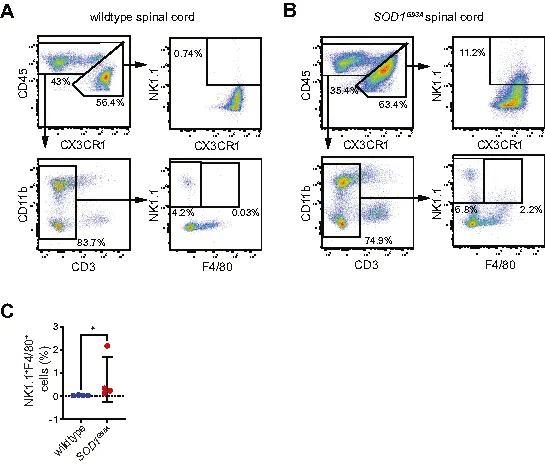
Supplementary Figure 5 legend.(**A**,**B**) Representative flow cytometry plots of spinal cord cells from 140-day-old wildtype (A) and *SOD1*^*G93A*^ (B) mice. Live CD45^+^ cells were gated using Zombie NIR viability dye (Fig. 3F). Within the CD45^+^ gate, cells were further resolved by CX3CR1 expression; NK1.1^+^ cells were identified from the CD45^int^CX3CR1^+^ resident microglial fraction. Within the CD45^high^CX3CR1^−^lymphoid cells and the CD45^high^CX3CR1^+^ CNS-associated macrophages, CD11b^+^CD3^−^ and CD11b^−^CD3^−^ cells were separately gated to identify NK1.1^+^F4/80^+^ cells. Numbers indicate percentage of gated populations. (**C**) Frequency of NK1.1^+^F4/80^+^ cells in the spinal cord of wildtype and *SOD1*^*G93A*^ mice. Data are mean ± SD; *P < 0.05 Mann-Whitney test.

**Figure:**
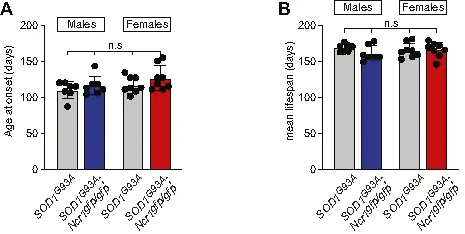
Supplementary Figure 6 legend.(**A**,**B**) Age at disease onset (A) and mean lifespan (B) of male and female *SOD1*^*G93A*^ and *SOD1*^*G93A*^*; Ncr1*^*gfp/gfp*^ mice. Data are means ± SD; n.s, non-significant; one-way ANOVA with Tukey-Kramer *post hoc*.

**Figure:**
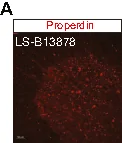
Supplementary Figure 7 legend (**A**) Representative image showing Properdin immunostaining (antibody LS-B13878) in the spinal cord of *SOD1*^*G93A*^ mice. Scale bar, 50 μm.

## Data Availability

Data will be made available on request.

## Abbreviations

ALS: Amyotrophic lateral sclerosis
ALSFRS-R: ALS functional rating scale
ChAT: Choline acetyltransferase
CNS: Central nervous system
GFAP: Glial fibrillary acidic protein
GFP: Green fluorescent protein
IL: Interleukin
ILC: Innate lymphoid cells
IFNγ: Interferon gamma
Klrb1: Killer cell lectin-like receptor subfamily B member 1
MHC: Major histocompatibility complex
NCR1: Natural cytotoxicity triggering receptor 1
NK: Natural killer
NKT: Natural killer T cell
SOD1: Cu-Zn superoxide dismutase
TNFα: Tumor necrosis factor alpha
Treg: Regulatory T cell

## Appendix A Supplementary data

Supplementary Table 1. Flow cytometry antibody panels. Multimedia component. 1
