# Supplementary material for: Limited contribution of natural killer cells to SOD1G93A ALS pathogenesis: evidence from comprehensive immunophenotyping and genetic deletion of Ncr1 and Properdin
Source: Brain Behav Immun Health. 2026 Nov 5;58:101401. doi: 10.1016/j.bbih.2026.101401 (PMC13763454; doi:10.1016/j.bbih.2026.101401)
Supplement: Supplementary file 1 [file mmc1.docx]

| **Figures** | **Tissue / Age** | **Markers** | **Population(s) / feature identified** |
| --- | --- | --- | --- |
| Suppl. Fig. 2A–E | Blood, 140 d | CD45, CD3, CD8, CD19 | CD3^+^CD8^+^ T cells, CD3^+^CD8^-^ T cells, CD19^+^ B lymphocytes (among CD45^+^ leukocytes) |
| Fig. 1A–C | Blood, 140 d | CD45, CD3, NK1.1, Ncr1 (antibody) or GFP (*Ncr1^gfp/gfp^* reporter) | Ncr1^+^NK1.1^+^ and GFP^+^NK1.1^+^ NK cells  (among CD45^+^CD3^−^ cells) |
| Fig. 1D–F | Blood, 140 d | CD45, CD3, CD49b, Ncr1 or GFP | Ncr1^+^CD49b^+^ and GFP^+^CD49b^+^ NK cells  (among CD45+CD3− cells) |
| Suppl. Fig. 3A–D | Blood & CNS, 150 d | CD45, CD90.2 (Thy1.2), CD3, CD49b | CD49b^+^ cells (among CD45^+^CD3^-^CD90.2^-^ cells) |
| Fig. 2A–D | Blood, 140 d | CD45, CD3, CD49b, CD11b, KLRG1,  Ncr1 or GFP | Stage D (CD49b^+^CD11b^-^KLRG1^-^),  stage E (CD49b^+^CD11b^+^KLRG1^−^) and  stage F (CD49b^+^CD11b^+^KLRG1^+^) NK cells  (among CD45^+^CD3^−^Ncr1^+^/GFP^+^ cells) |
| Fig. 2E,F | Blood, 140 d | NK1.1, CD27, Ncr1 or GFP | Stage C-E (CD27^+^NK1.1^+^) NK cells (among Ncr1^+^/GFP^+^ cells) |
| Fig. 2G,H | Blood, 140 d | NK1.1, CD69, Ncr1 or GFP | Activated (CD69^+^NK1.1^+^) NK cells  among Ncr1^+^/GFP^+^ cells) |
| Suppl. Fig. 4 | Blood, 110 d | CD45, CD3, NK1.1, CD11b, KLRG1 | NK1.1^+^ among CD45^+^CD3^-^ cells, CD11b^+^KLRG1^-^ and CD11b^+^KLRG1^+^ among NK1.1+ cells |
| Fig. 3C-E | Spinal cord, 110 d | CD45, CD3, NK1.1, Ncr1 or GFP | Ncr1^+^NK1.1^+^ and GFP^+^NK1.1^+^ cells  (among CD45^+^CD3^−^ cells) |
| Fig. 3F-I | Spinal cord, 140 d | CD45, CD3, NK1.1, CX3CR1 | CD45^+^CD3^-^NK1.1^+^, CD45^+^CD3^+^NK1.1^+^and CD45^int^NK1.1^+^CX3CR1^+^ cells |
| Suppl. Fig. 5 | Spinal cord, 140 d | CD45, CD3, CX3CR1, NK1.1, CD11b, F4/80 | NK1.1^+^F4/80^+^ cells (among CD45^high^CX3CR1^+/-^CD11b^+/-^CD3^-^ cells) |
